# 

**Published:** 1867-07

**Authors:** 


### Books and Pamphlets Received.

A Practical Guide to the study of the Diseases of the Eye—their Medical and Surgical Treatment. By Henry W. Williams, M. D., Cantab., Ophthalmic Surgeon to the City Hospital, Boston; University Lecturer on Ophthalmic Surgery in Harvard University, etc. Second edition, revised and enlarged. Boston: Ticknor & Fields, 1867. Breed, Lent & Co., Buffalo.

Menstruation; or, the Menstrual Flow; an Epiphenomenon of Ovulation. An Argumental Treatise, read before the St. Louis Medical Society, on the question: Is Menstruation Ovulation? By G. M. B. Maughs, M. D., Professor of Obstetrics and Diseases of Women and Children in the Humboldt Medical College, St. Louis, Mo.

Quarterly Summary of the Transactions of the College of Physicians of Philadelphia, from December 6, 1865 to November 7, 1866, inclusive.

Twenty-fourth Annual Report of the Managers of the New York State Lunatic Asylum, for the year 1866.

Annual Circular and Catalogue of the Bellevue Hospital Medical College, City of New York, 1867–68.

Annual Circular of the Trustees and Faculty of the Medical College of the State of South Carolina, with a Catalogue of Students and List of Graduates, Session 1866–67.

Second Annual Announcement of the Humboldt Medical College, St. Louis, Mo., 1867–68.

Thirty-first Annual Announcement of the Medical Department of the University of Louisville, Session of 1867–68, with a Catalogue of Students for Session 186–7.

